# Reduced hippocampal gray matter volume is a common feature of patients with major depression, bipolar disorder, and schizophrenia spectrum disorders

**DOI:** 10.1038/s41380-022-01687-4

**Published:** 2022-07-15

**Authors:** Katharina Brosch, Frederike Stein, Simon Schmitt, Julia-Katharina Pfarr, Kai G. Ringwald, Florian Thomas-Odenthal, Tina Meller, Olaf Steinsträter, Lena Waltemate, Hannah Lemke, Susanne Meinert, Alexandra Winter, Fabian Breuer, Katharina Thiel, Dominik Grotegerd, Tim Hahn, Andreas Jansen, Udo Dannlowski, Axel Krug, Igor Nenadić, Tilo Kircher

**Affiliations:** 1 Department of Psychiatry and Psychotherapy, Philipps-University Marburg, University Hospital Marburg, UKGM, Marburg, Germany; 2 Center for Mind, Brain and Behavior (CMBB), Marburg, Germany; 3 Department of Psychiatry, Social Psychiatry and Psychotherapy, Hannover Medical School, Hannover, Germany; 4 Core-Facility BrainImaging, Faculty of Medicine, Philipps-University Marburg, Marburg, Germany; 5 Institute for Translational Psychiatry, University of Münster, Münster, Germany; 6 Institute for Translational Neuroscience, University of Münster, Münster, Germany; 7 Department of Psychiatry and Psychotherapy, University of Bonn, Bonn, Germany

**Keywords:** Neuroscience, Psychiatric disorders

## Abstract

Major depressive disorder (MDD), bipolar disorder (BD), and schizophrenia spectrum disorder (SSD, schizophrenia, and schizoaffective disorder) overlap in symptomatology, risk factors, genetics, and other biological measures. Based on previous findings, it remains unclear what transdiagnostic regional gray matter volume (GMV) alterations exist across these disorders, and with which factors they are associated. GMV (3-T magnetic resonance imaging) was compared between healthy controls (HC; *n* = 110), DSM-IV-TR diagnosed MDD (*n* = 110), BD (*n* = 110), and SSD patients (*n* = 110), matched for age and sex. We applied a conjunction analysis to identify shared GMV alterations across the disorders. To identify potential origins of identified GMV clusters, we associated them with early and current risk and protective factors, psychopathology, and neuropsychology, applying multiple regression models. Common to all diagnoses (vs. HC), we identified GMV reductions in the left hippocampus. This cluster was associated with the neuropsychology factor working memory/executive functioning, stressful life events, and with global assessment of functioning. Differential effects between groups were present in the left and right frontal operculae and left insula, with volume variances across groups highly overlapping. Our study is the first with a large, matched, transdiagnostic sample to yield shared GMV alterations in the left hippocampus across major mental disorders. The hippocampus is a major network hub, orchestrating a range of mental functions. Our findings underscore the need for a novel stratification of mental disorders, other than categorical diagnoses.

## Introduction

Given that mental disorders such as major depressive disorder (MDD), bipolar disorder (BD), and schizophrenia spectrum disorder (SSD) exhibit high overlap in many domains (e.g., psychopathology, genetics, neuropsychology, regional brain volume alterations), the research question has emerged whether they are characterized by more communalities rather than differences [1–6]. In the domain of brain morphometry, previous work has attempted, but failed, to find a unique neural correlate (i.e., biomarker) specific to one such disorder [2].

Gray matter volume (GMV) changes have been described in single case vs. control studies: In MDD, GMV reductions were reported in the subgenual cingulate cortex, hippocampus, amygdala, thalamus, fusiform gyrus and putamen [7–9]. In BD, reductions were found in the anterior cingulate cortex (ACC), superior temporal gyrus, and calcarine cortices [10]. In schizophrenia (SZ; and schizoaffective disorder (SZA), referred to as SSD), reductions were reported in the frontal opercula, insula, superior temporal, mid cingulate, and calcarine cortices, cerebellum, hippocampus, amygdala, and basal ganglia [10, 11]. However, within these diagnostic categories, studies have produced heterogeneous results, which have been attributed to differences in course of illness, medication, age of onset, risk factors, genetics, psychopathology, and comorbidities, among others [7, 9, 12–15].

To disentangle these heterogeneous intra- and interdiagnostic findings, a landmark meta-analysis across six mental disorders pooled *N* = 193 original neuroimaging studies with *N* = 15,892 subjects [2]. Here, transdiagnostic GMV reductions in all patients vs. controls were identified in the bilateral insulae, right thalamus, left subgenual ACC, right dorsal and ventral ACC/mPFC, left amygdala, and left hippocampus. This study included a wide range of psychiatric disorders: MDD, BD, SZ, addiction, anxiety, and obsessive-compulsive disorder.

To answer the question which GMV alterations specifically overlap and are distinct in MDD, BD, and SSD –– that is, disorders of the affective and psychotic spectrum, a direct comparison of large, matched patient groups is necessary.

Thus far, only one study directly compared GMV brain structural alterations in MDD, BD, and SZ [16]. The authors found that almost 88% of GMV reductions overlapped in all three diagnostic groups, including regions in the temporal pole, orbitofrontal cortex (OFC), insula, hippocampus, cingulate and angular gyri. However, this study only included adolescents and young adults (aged 13–30), not matched for age and sex [16].

Since there is first evidence for significant GMV overlap across affective and psychotic disorders [2], it raises the question whether there are common, underlying factors that are responsible for these transdiagnostic findings. From previous structural brain imaging research, we know that the following four domains, among others, have been associated with brain structure in patients and healthy controls (HC): (1) early risk and protective factors: childhood maltreatment, urbanicity, familial risk, gestational age, birth weight, and parental bonding [12, 17–20], (2) current risk and protective factors: life events, resilience, and social support [21–23], (3) psychopathology: global functioning, positive, negative, depressive, and manic symptoms [3, 24], and (4) neuropsychological performance [25]. To the best of our knowledge, it has not been investigated whether transdiagnostic morphometric alterations are related to transdiagnostic phenotypical or risk patterns. However, such investigations are crucial as they could shed light on potential transdiagnostic pathomechanisms or indicate starting points for prevention.

In summary, previous studies on GMV alterations across MDD, BD and SSD have several shortcomings: (1) only comparing two diagnostic groups, (2) small sample sizes, (3) not matching groups for age and sex, and (4) not relating transdiagnostic brain alterations to phenotypical or risk factors. To overcome these issues, we directly compared three large age and sex-matched diagnostic groups (MDD, BD, SSD) to HC, drawn from a cohort of *n* = 1927, to identify shared and distinct GMV alterations. Further, we associated morphometric alterations with phenotypic and risk factors, i.e., early risk and protective factors, current risk and protective factors, psychopathology, and neuropsychological performance.

## Methods

### Participants

For the current study, from a cohort of *n* = 1927, we selected an age and sex-matched sample of *N* = 440 with *n* = 110 acute, chronic, and remitted participants per group of MDD, BD, SSD, and HC (matched using the MatchIt package [26] in R [27]) (see Table 1 for descriptive statistics). As the SSD group was the smallest (*n* = 110), MDD and BD patients and HCs were matched 1:1 to the age and sex-distribution of the SSD group. Participants aged 18–65 were drawn from the Marburg-Münster Affective Disorder Cohort Study (MACS). MACS is a longitudinal bi-center cohort study, and part of FOR2107, a consortium investigating the neurobiology of major psychiatric disorders [28].

**Table 1:** Descriptive statistics.

	HC (*n* = 110)	MDD (*n* = 110)	BD (*n* = 110)	SSD (*n* = 110)	*p*
Age	38.83 (12.21)	38.35 (11.7)	39.81 (12.25)	38.37 (11.71)	0.783
Sex	*M* = 54	*M* = 56	*M* = 51	*M* = 56	0.736
	*F* = 56	*F* = 54	*F* = 59	*F* = 54	
TIV	1529.98 (146.74)	1520.97 (172.35)	1539.99 (137.63)	1542.78 (180.99)	0.566
Age of onset	–	27 (12.19)	23.47 (10.37)	22.51 (9.02)	0.005^a^
In-patient treatment *n*, (%)	–	40 (36.36%)	28 (25.45%)	48 (43.64%)	0.012
Remission status^h^	–	*A* = 65	*A* = 69	*A* = 75	0.371
		*R* = 45	*R* = 41	*R* = 35	
Antidepressant *n*, (%)	–	71 (64.55%)	46 (41.82%)	32 (29.09%)	<0.001
Antipsychotics *n*, (%)	–	21 (19.09%)	50 (45.45%)	95 (86.36%)	<0.001
Lithium n, (%)	–	2 (1.82%)	31 (28.18%)	4 (3.64%)	<0.001
Anticonvulsive *n*, (%)	–	2 (1.82%)	36 (32.73%)	8 (7.27%)	<0.001
Comorbidity *n*, (%)	–	37 (33.64%)	46 (41.82%)	42 (39.09%)	0.453
HAMA	2.09 (2.61)	12.69 (8.15)	9.25 (7.35)	9.63 (7.37)	<0.001^b^
HAM-D	1.0 (1.6)	7.65 (5.94)	6.5 (5.97)	6.55 (5.78)	<0.001^c^
SANS	0.87 (2.24)	7.04 (7.52)	5.34 (7.03)	12.73 (12.52)	<0.001^d^
SAPS	0.20 (0.71)	0.38 (1.18)	2.3 (4.38)	10.6 (13.26)	<0.001^e^
YMRS	0.47 (1.06)	1.13 (1.77)	3.89 (5.94)	2.99 (5.12)	<0.001^f^
GAF	91.43 (7.4)	63.89 (13.7)	62.81(12.75)	54.93 (12.39)	<0.001^g^

Mean (standard deviation); *TIV* total intracranial volume, *HAMA* Hamilton anxiety rating scale, [41] *HAM-D* Hamilton rating scale for depression, [40] *SANS* scale for the assessment of negative symptoms, [38] *SAPS* scale for the assessment of positive symptoms, [39] *YMRS* Young mania rating scale, [42] *GAF* global assessment of functioning [29] *A* acute, *R* partially or fully remitted.

Post-hoc comparisons between groups (Tukey´s post-hoc test):

^a^MDD > BD, SSD.

^b^HC < MDD, BD, SSD; MDD > BD, SSD.

^c^HC < MDD, BD, SSD.

^d^HC < MDD, BD, SSD; SSD > MDD, BD.

^e^SSD > HC, MDD, BD.

^f^HC < MDD, BD, SSD; BD > SSD, MDD; SSD > MDD.

^g^HC > MDD, BD, SSD; MDD > SSD; BD > SSD.

^h^Remission data were missing for *n* = 76 patients.

Participants were recruited from in and out-patient departments of the universities of Marburg and Münster, Germany, local psychiatric hospitals (Vitos Marburg, Gießen, Herborn, and Haina, LWL Münster, Germany), and via postings in local newspapers and flyers. During a semi-structured interview, the German version of the structured clinical interview (SCID-I) for the DSM-IV-TR was applied by trained staff [29]. Furthermore, within one week of MRI scanning, psychopathological scales, a large questionnaire battery, a neuropsychological test battery and other rater-based scales were applied (see below). Moreover, clinical variables such as age of onset and number of hospitalizations were assessed during the semi-structured interview and, if available, based on patient records.

Exclusion criteria were a history of neurological or general medical conditions, current or lifetime alcohol dependency, current substance dependency, or current use of benzodiazepines (all assessed during the semi-structured interview performed by trained personnel and via self-report questionnaires), and verbal IQ ≤ 80 (assessed using [30]). Further exclusion criteria for the HC group were current or past mental disorders according to DSM-IV-TR, and lifetime intake of psychotropic medication. The study protocols were approved by the local Ethics Committees of Marburg and Münster, Germany according to the Declaration of Helsinki. All participants gave written informed consent before participation and received financial compensation.

### Transdiagnostic phenotypical domains

To associate GMV with phenotype and risk factors, four domains were investigated: (1) early risk and protective factors, (2) current risk and protective factors, (3) psychopathology, (4) neuropsychological performance.

### Early risk and protective factors

Childhood maltreatment was assessed using the childhood trauma questionnaire (CTQ) [31], urbanicity was assessed using a score for urban upbringing [32]. Familial risk was assessed via a questionnaire, asking whether a first-degree relative had been diagnosed and/or treated for MDD, BD, or SSD. Gestational age and birth weight were assessed through a questionnaire. Maternal and paternal care as an early protective factor was assessed with the German version of the parental bonding instrument (PBI) [33, 34].

### Current risk and protective factors

Stressful life events (during the last six months) and their subjective impact were assessed using the life event questionnaire (LEQ) [35]. Current protective factors such as social support and resilience were assessed using the social support questionnaire (FSozu) [36] and the RS-25 resilience questionnaire, respectively [37].

### Psychopathology

Psychopathology was assessed using the Scale for the Assessment of Negative Symptoms (SANS) [38], the Scale for the Assessment of Positive Symptoms (SAPS) [39], the Hamilton Depression Scale (HAM-D) [40], the Hamilton Anxiety Scale (HAMA) [41], and the Young Mania Rating Scale (YMRS) [42]. Further, the Global Assessment of Functioning (GAF) score [43, 44], was applied. All scales were rater-based. Raters were trained in the evaluation of psychopathological symptoms. Interrater reliabilities (ICC) were >0.86 for all scales.

### Neuropsychological performance

A comprehensive neuropsychological test battery was applied including the d2 attention test [45], verbal fluency [46], symbol-coding [47], spatial span [48], letter-number span [49], Trail-Making Test A and B (TMT) [50], and the German Verbal Learning and Memory Test (VLMT) [51].

### MRI data acquisition and preprocessing

MRI data acquisition and preprocessing was performed according to standardized procedures and using default parameters implemented in the respective toolboxes. At both sites, a 3-T MRI scanner (Marburg: Tim Trio, Siemens, Germany; Münster: Prisma, Siemens, Germany) was used to acquire T1 weighted images using a fast gradient echo MP-RAGE sequence with a slice thickness of 1.0 mm and a field of view of 256 mm. In Marburg, a 12-channel head matrix Rx-coil was used, in Münster, a 20-channel head matrix Rx-coil was used. Parameters differed across sites: Marburg: 176 sagittal slices, time of repetition (TR) = 1.9 s, time of echo (TE) = 2.26 ms, inversion time (TI) = 900 ms, flip angle = 9°; Münster: 192 sagittal slices, TR = 2.13 s, TE = 2.28 ms, TI = 900 ms, flip angle = 8°. MRI data were acquired according to an extensive quality assurance protocol [52].

A senior clinician visually inspected all scans regarding artifacts and anatomical abnormalities before preprocessing. Structural MRI data were preprocessed with the CAT12-Toolbox (Computational Anatomy Toolbox for SPM, build 1720, Structural Brain Mapping group, Jena University Hospital, Germany) (http://dbm.neuro.uni-jena.de/cat/) building on SPM12 (Statistical Parametric Mapping, Institute of Neurology, London, UK) using default parameters. In short, preprocessing included image segmentation into gray matter, white matter, and cerebrospinal fluid [53], spatial registration, and normalization [54]. Data were normalized to Montreal Neurological Institute (MNI) space. A more detailed description of our quality protocol can be found elsewhere [52]. During preprocessing, total intracranial volume (TIV) was calculated. MRI data sets were spatially smoothed with a Gaussian kernel of 8 mm full width half maximum.

### Statistical analyses

#### Factor analysis of neuropsychological tests

Using SPSS 27, (Statistical Package for Social Science, IBM) an explorative principal axis factor analysis with varimax rotation was performed to identify aggregated domains of cognitive functioning (see Supplement). As neuropsychological test variables were differentially scaled, z-transformed values were used for the factor analysis. Suitability of neuropsychological test data for factor analysis was tested using Bartlett’s test of sphericity [55] and the Kaiser–Meyer–Olkin test (KMO) [56]. Factors were extracted according to the Kaiser’s eigenvalue greater than one criterion [57]. Factor loadings were extracted using the regression method as implemented in SPSS.

#### Brain structural analysis

To identify differences across the four groups (HC, MDD, BD, SSD), smoothed GMVs were applied using a whole brain 1×4 design in SPM (v6906) running under Matlab (R2017a). Post-hoc tests between two respective groups were performed to investigate significant differences. To identify commonly altered areas across the three diagnoses vs. healthy participants, we performed a conjunction analysis. Conjunction analysis allows the identification of overlap in GMV alterations in single diagnosis vs. control comparisons. The conjunction analysis used here was defined as follows: HC > MDD ∩ HC > BD ∩ HC > SSD. As conjunction analysis is known to be conservative, we opted for a region of interest (ROI)-based approach which included previously identified ROIs reported in the meta-analysis [2] in MDD, BD, and SSD patients by Goodkind et al. (2015) [2]. These ROIs were the bilateral insulae, thalamus, ACC, left amygdala, and left hippocampus. Using the Dartel space neuromorphometrics atlas in CAT12, we created one mask that included these anatomical ROIs. We have also included an exploratory whole-brain conjunction analysis in the Supplement.

The covariates age, sex, and total intracranial volume (TIV) were used in all analyses. MRI data were acquired at two sites. As recommended by the MRI quality assurance protocol of the FOR2107 cohort, we used two dummy-coded variables accounting for the change of a body coil and site (Marburg pre body coil: yes/no, Marburg post body coil: yes/no, and Münster as reference category [52]). Following the CAT12 recommendations (http://dbm.neuro.uni-jena.de/cat/), threshold masking with a value of 0.1 was applied for all analyses to exclude non-brain areas. Results were considered significant at *p* < 0.05 cluster-level family wise error-corrected (FWE) for multiple comparisons after an initial threshold of *p* < 0.001 uncorrected, with a cluster extend threshold of *k* > 10. Significant clusters were labelled using the Dartel space Neuromorphometrics atlas (http://www.neuromorphometrics.com/).

#### Transdiagnostic phenotypical factors associated with GMV

To answer the question which factors were associated with transdiagnostic morphometric findings, we associated the identified conjunction cluster with four different domains. These were (1) early risk and protective factors, (2) current risk and protective factors, (3) psychopathology and (4) neuropsychology (see above). To this end, we extracted eigenvariate values (weighted means) as an approximation of mean value from the conjunction cluster. Using SPSS 27, we ran four separate multiple regression models. We decided against one combined model, as this would have resulted in a largely reduced sample size. Using four models allowed us to minimize sample size loss without having to impute values. In all models, age, sex, TIV, two site/bodycoil variables, and a group variable were used as covariates. Multicollinearity in these variables was absent.

The first model, early risk and protective factors, included childhood maltreatment, urbanicity, parental bonding, gestational age, birth weight, and familial risk. The second model, current risk and protective factors, included current life events, resilience, and social support. The third model, psychopathology, was comprised of global functioning, positive, negative, depressive, and manic symptomatology. The fourth model, neuropsychological performance, included three factors identified in the factor analysis described in the Supplement.

## Results

Our analyses were subdivided into four steps: (1) investigation of global effects of GMV (F-Test), (2) investigation of post-hoc two-group comparisons of GMV (*t*-Tests), (3) conjunction analysis of HC > MDD ∩ HC > BD ∩ HC > SSD, and (4) association of the conjunction cluster with phenotypical domains. Additional analyses controlling for the effect of medication and comorbidity showed that results remained stable. These analyses can be found in the Supplement.

To rule out that group differences were driven by HCs, we re-ran all analyses including *n* = 220 more HCs, resulting in a total sample of *N* = 660. These analyses revealed a similar pattern to the original analyses and can be found in the Supplement.

### Global effects of GMV (F-Test)

**Fig. 1:**
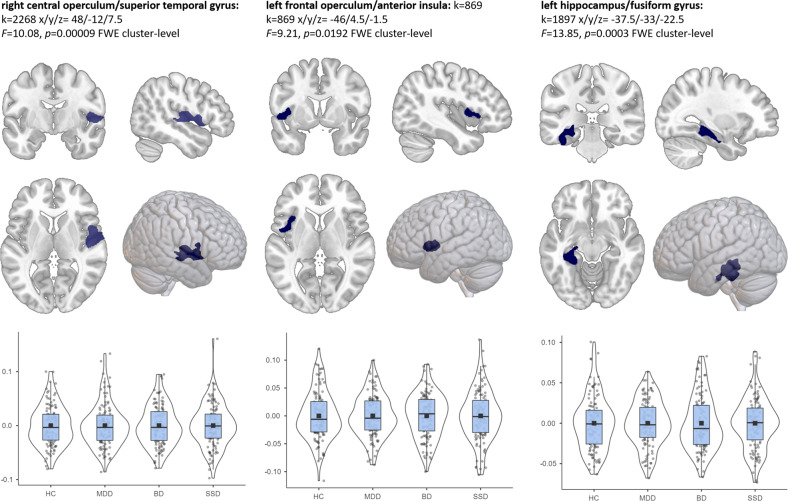
Significant clusters exhibiting GMV differences between HC, MDD, BD, and SSD (F-Test). Combined boxplots and violin plots show value distribution for the groups in the respective cluster.

For the four groups (HC, MDD, BD, SSD), three significant clusters emerged in the *F*-Test. The first cluster comprised parts of the left fusiform gyrus, hippocampus, parahippocampal, inferior medial temporal, and lingual gyri (*k* = 1897, x/y/z = −37.5/−33/−22.5), *F* = 13.85, *p* = 0.0003 FWE cluster-level; *k* = 69, *p* = 0.0004 FWE peak-level). The second cluster comprised the right central operculum, superior temporal gyrus, temporal transverse gyrus, planum polare, and the precentral gyrus (*k* = 2268, x/y/z = 48/−12/7.5), *F* = 10.08, *p* = 0.00009 FWE cluster-level). The third cluster comprised parts of the left central and frontal operculum, anterior insula, planum polare, and temporal pole (*k* = 869, x/y/z = −46.5/4.5/−1.5), *F* = 9.21, *p* = 0.0192 FWE cluster-level) (cf. Fig. 1). To exclude the potential influence of duration and severity of illness on the identified clusters, we investigated the association between each cluster with number of hospitalizations, duration of hospitalization, duration in manic/depressive/psychotic state, and age of onset. There was no significant association between any of these factors and any cluster (all *p*s > 0.05), highlighting that the identified clusters were independent of duration and severity of illness.

### Investigation of post-hoc two-group comparisons of GMV (*t*-Tests)

**Fig. 2:**
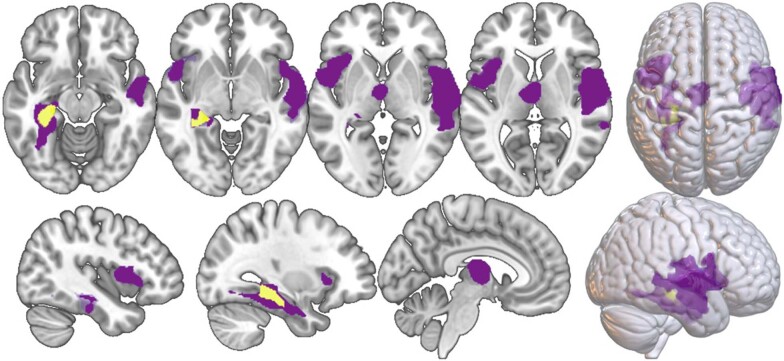
Post-hoc comparisons of healthy controls - patients. HC > SSD clusters are presented in purple; HC > MDD cluster is presented in yellow.

**Fig. 3:**
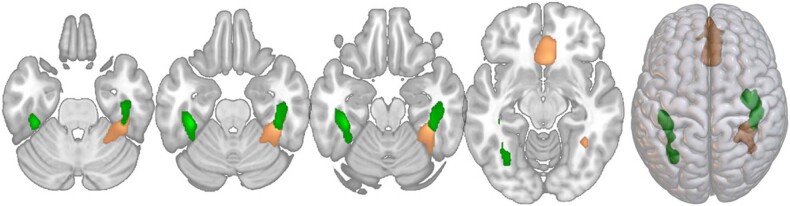
Post-hoc within patient comparisons. GMV differences were present for MDD > SSD (orange) and BD > SSD (green); for details see Table 2.

Next, we investigated which two-group comparisons were driving the clusters of the *F*-Test. Here, several significant areas (FWE cluster-level corrected) emerged for specific patient–HC comparisons, and within patient group comparisons. Table 2 lists all clusters, and Figs. 2 and 3 visualize these significant clusters in patient–HC comparisons and within patient comparisons, respectively.

**Table 2:** Results of FWE-cluster level significant post-hoc t-tests.

		MNI coordinates					
	H	x	y	z	*t*	*k* cluster	*p* FWE cluster level
HC vs. diagnostic group							
HC > MDD							
35% hippocampus	L	−30	−31.5	−12	4.63	1554	0.033
15% parahippocampal gyrus							
14% fusiform gyrus							
HC > SSD							
29% superior temporal gyrus	R	63	−1.5	4.5	5.31	8239	8.29*10^−9 a^
19% central operculum							
17% middle temporal gyrus							
30% central operculum	L	−46.5	4.5	−1.5	5.23	2315	0.0009^b^
27% frontal operculum							
25% anterior insula							
41% fusiform gyrus	L	−37.5	−31.5	−13.5	4.95	3420	6.27*10^−5 c^
15% hippocampus							
11% parahippocampal gyrus							
76% thalamus proper	B	−3	−12	15	4.85	1708	0.004^d^
Differences between diagnostic groups							
MDD < BD							
34% medial orbital gyrus	R	9	21	−10.5	4.45	961	0.041
28% subcallosal area							
21% anterior cingulate gyrus							
MDD > SSD							
78% fusiform gyrus	L	−37.5	−33	−24	5.81	1196	0.02^e^
11% occipital fusiform gyrus							
10% inferior temporal gyrus							
69% fusiform gyrus	R	36	−13.5	−34.5	4.59	1695	0.006
31% inferior temporal gyrus							
BD > SSD							
68% fusiform gyrus	R	37.5	−37.5	−21	4.88	1769	0.004^f^
24% exterior cerebellum							
26% medial frontal cerebrum (R)	B	6	24	−12	4.67	1671	0.005^g^
19% anterior cingulate gyrus (B)							
18% superior medial frontal gyrus (R)							

Only areas ≥10% are included. Letters indicate clusters which were also significant at FWE peak level.

*H* hemisphere, *R* right, *L* left, *B* bilateral.

^a^also significant at *p* < 0.05 FWE peak-level corrected, *k* = 571, *p* = 0.002.

^b^also significant at *p* < 0.05 FWE peak-level corrected, *k* = 164, *p* = 0.003.

^c^also significant at *p* < 0.05 FWE peak-level corrected, *k* = 81, *p* = 0.009.

^d^also significant at *p* < 0.05 FWE peak-level corrected, *k* = 41, *p* = 0.015.

^e^also significant at *p* < 0.05 FWE peak-level corrected, *k* = 113, *p* = 0.0001.

^f^also significant at *p* < 0.05 FWE peak-level corrected, *k* = 48, *p* = 0.003.

^g^also significant at *p* < 0.05 FWE peak-level corrected, *k* = 19, *p* = 0.031.

### Common areas of GMV alterations across MDD, BD, and SZ

#### ROI analyses

**Fig. 4:**
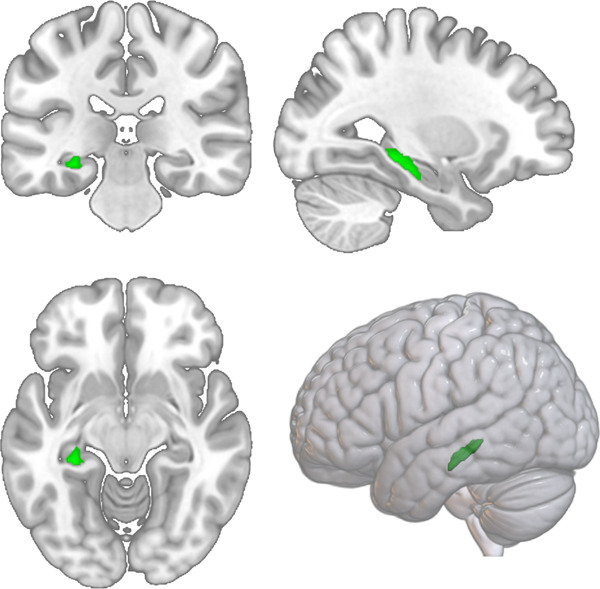
Hippocampal volume reductions in all patients compared to healthy controls. *Results from the conjunction analysis (*HC > MDD ∩ HC > BD ∩ HC > SSD): GMV reductions common in all diagnoses compared to HC in the left hippocampus, (k = 316, x/y/z = −30/−33/−10.5), *T* = 4.25, *p* = 0.042 FWE cluster level.

To investigate overlapping GMV reductions in all patients compared to HC, we ran a conjunction analysis (*HC* > *MDD* ∩ *HC* > *BD* ∩ *HC* > *SSD)* using the ROIs from the meta-analysis by Goodkind et al., (2015). This analysis revealed GMV reductions in the patient groups in the left hippocampus (*k* = 316, x/y/z = −30/−33/−10.5), *T* = 4.25, *p* = 0.042 FWE cluster-level; *k* = 316, *p* = 0.01 FWE peak-level) (see Fig. 4).

#### Whole-brain analyses

Results of the exploratory whole-brain conjunction analysis can be found in Supplement 3, revealing a similar pattern.

### Association of conjunction cluster with phenotypical domains

Lastly, we associated the conjunction cluster in the left hippocampus with four phenotypic and risk domains using multiple regressions.

#### Early risk and protective factors

The first model included early risk and protective factors previously associated with GMV in mental disorders. The model significantly predicted hippocampal volume, *F*(12, 190) = 12.89, *p* < 0.001, *R*^*2*^ = 0.78, (adj. *R*^*2*^ = 0.57). Only the covariates age (β = −0.141, *p* = 0.004), TIV (*β* = 0.68, *p* < 0.001) and the group covariate (*β* = −0.225, *p* < 0.001) were significant predictors. Early risk and protective factors (childhood maltreatment, urbanicity, birth weight, gestation age, familial risk, parental bonding, site) were no significant predictors for hippocampal GMV (all *p*s > 0.05).

#### Current risk and protective factors

The second model included current risk and protective factors associated with GMV in mental disorders. The model significantly predicted hippocampal volume, *F*(9, 413) = 60.66, *p* < 0.001, *R*^*2*^ = 0.57, (adj. *R*^*2*^ = 0.56). LEQ score was a significant predictor (*β* = −0.071, *p* = 0.035), along with the covariates TIV (*β* = 0.673, *p* < 0.001), age (*β* = −0.172, *p* < 0.001), and group (*β* = −0.201, *p* < 0.001). Resilience, social support, sex, and the site variables were not significant (all *p*s > 0.05).

#### Psychopathology

The third model included positive, negative, depressive, anxious, and manic symptomatology assessed with the sum scores of several psychopathological scales. The total model significantly predicted hippocampal volume, *F*(12, 427) = 46.11, *p* < 0.001, *R*^*2*^ = 0.56, (adj. *R*^*2*^ = 0.55). GAF was a significant predictor for hippocampal volume (*β* = 0.142, *p* = 0.017). None of the other psychopathology variables, i.e., positive, negative, depressive, anxiety, and manic symptoms (SANS, SAPS, HAMD, HAMA, YMRS), and neither the site variables, nor sex were significant predictors of hippocampal volume (all *p*s > 0.05). The covariates group (*β* = −0.111, *p* = 0.027), TIV (*β* = 0.665, *p* < 0.001), and age (*β* = −0.162, *p* < 0.001) significantly predicted hippocampal GMV.

#### Neuropsychological performance

Using exploratory factor analysis, we identified three factors of neuropsychological performance: F1: working memory/executive functioning, F2: verbal fluency, and F3: verbal episodic memory. A detailed description can be found in Supplement 1.

The model including the neuropsychological domain significantly predicted hippocampus volume *F*(9, 404) = 56.58, *p* < 0.001, *R*^*2*^ = 0.56, (adj. *R*^*2*^ = 0.55). The neuropsychological performance factor working memory/executive functioning was a significant predictor (*β* = 0.10, *p* = 0.009), together with the covariates: group (*β* = −0.20, *p* < 0.001), TIV (*β* = 0.67, *p* < 0.001), and age (*β* = −0.117, *p* = 0.001). The variables for site, sex, verbal fluency, and *verbal episodic memory* were no significant predictors (all *p*s > 0.05).

In sum, the transdiagnostic left hippocampal GMV alteration was significantly negatively associated with stressful life events and positively with global functioning and working memory/executive functioning, independent of the presence of a psychiatric disorder.

## Discussion

The central goal of this study was the identification and characterization of transdiagnostic GMV alterations common to MDD, BD, and SSD. We identified transdiagnostically reduced left hippocampal GMV, which was associated with stressful life events, global functioning, and executive functioning. We discuss these finding and further group comparisons below.

### Group differences

Group differences between HC, MDD, BD, and SSD were detected in three main clusters: insula, superior temporal gyrus (STG), and hippocampus. Post-hoc group comparisons revealed significant GMV differences in the insula and STG only in SSD compared to HC. Further, SSD patients showed reduced GMV in the fusiform gyrus compared to HC, MDD, and BD.

Our findings align with previous research and expand them: The relevance of insula function in psychosis, especially with regard to aberrant information processing and integration, has been discussed extensively [2, 10, 58–63]. Reviewing findings of the STG in schizophrenia, Lu et al. (2009) report its involvement particularly in formal thought disorder and hallucinations [64].

GMV in the fusiform gyrus in SSD patients have also been documented and associated with reduced ability for facial recognition [65]. Our study further demonstrated that fusiform gyrus volume was reduced in SSD patients compared to MDD and BD patients, as well as HC, indicating a diagnosis specific effect. Recent studies have further associated fusiform gyrus volume with the paranoid-hallucinatory syndrome and the positive symptoms [3, 66].

### Diagnoses-common GMV alterations

Hippocampal volume reductions have been documented in case control studies of MDD, BD, and SZ [7, 14, 67], and in meta-analyses [2, 68, 69], although with inconsistencies within single diagnoses. Hippocampal reductions in patients have been explained through heightened stress, leading to dysregulation of the hypothalamic-pituitary-adrenal (HPA) axis and increased production of glucocorticoids, which have neurotoxic effects [70]. To explore this volume loss, we investigated associations with potential phenotypic and risk and protective factors. We found smaller left hippocampal volume to be associated with higher number of stressful life events, poorer neuropsychological executive and global (social) functioning (GAF), but notably not to other potential factors which have previously been associated with hippocampal volume, e.g., childhood maltreatment or psychopathology [71, 72]. Associations between hippocampal volume and childhood maltreatment have been heterogeneous, with some studies reporting a significant negative association between these factors [71, 73], whereas others did not [15]. A meta-analysis highlighted the importance of age, sex, and diagnosis, as they moderate this association [74].

The association between stressful life events and reduced hippocampal volume has been reported in a longitudinal study [75], and in the context of 9/11 [76]. We demonstrated that the number and impact of stressful life events was associated with reduced hippocampal volume in both patients and healthy subjects. We further demonstrated that GAF score and the neuropsychological performance factor working memory/executive functioning were associated with hippocampal volume. These findings align with results from case control studies, particularly in SZ, and less consistently in affective disorders [77–81].

While previous studies had linked verbal memory to hippocampal volume, our findings show associations with working memory/executive function, which has also been documented in several previous studies (e.g [80, 82–84]). However, our results go beyond these findings, as we demonstrated that these effects are not related to the traditional, descriptive diagnoses, but apply to all patients and healthy controls on a dimensional level. Reduced left hippocampal volume in all patients mirrors findings from molecular genetics [1, 85] and imaging studies [2, 16], highlighting that psychiatric disorders share large biological overlap across disorders.

Intriguingly, electroconvulsive therapy (ECT) has been identified as a potential intervention to *increase* hippocampal volume, global functioning, and quality of life in patients [86–88]. We believe these results emphasize the validity of hippocampal GMV as a transdiagnostic correlate of psychopathology that could be a target for intervention and prevention.

We provided a large, matched sample, making our findings more robust and generalizable compared to previous work [2, 16]. First, matched samples are adjusted for effects of confounding variables between groups [26]. Second, we provided data from patients aged 18–65, thus allowing for more generalizable results. Findings from another study (with patients aged 13–30) [16] might be skewed towards early-onset patients, who might represent a distinct subgroup, as median age of onset for MDD and BD is later in life (30–33 years) [89–91].

### Limitations

Some limitations should be noted. First, we did not detect a significant whole-brain difference in the HC > BD contrast but detected a significant hippocampus finding in the conjunction analysis using ROI analysis. Meta- and mega-analyses have demonstrated that lithium medication increases volume in the hippocampus in BD patients [69, 92]. This factor seems to have impeded the detection of a whole-brain effect in BD vs. HC. In their meta-analysis, Hajek and colleagues (2012) [69] described this effect and were able to demonstrate that lithium merely masked the effect of actual hippocampal volume loss in BD patients. Second, while psychiatric medication is known to potentially affect GMV, the inclusion of all psychiatric medication as a covariate has inherent limitations in transdiagnostic samples. Cumulative lifetime intake of psychiatric medication, in particular antipsychotics, has been associated with GMV. However, it is difficult to reliably obtain this information, and we only had information on current medication available. Correcting for medication remains a challenge in transdiagnostic imaging studies: While it might be possible to only include medication naïve patients, this would in turn result in restricted representativeness. In the present study, we covered the full spectrum of patients including a broad age range, duration of illness, severity, and comorbidities. Most importantly, psychopharmacological medication class (antipsychotics, antidepressants, etc.) is highly intercorrelated with DSM diagnoses and illness severity and can therefore not entirely be eliminated in statistical models [93, 94]; inclusion of medication as a covariate would have drastically reduced variance to detect differences in group GMV, and we therefore decided not to include it in the main analyses, in line with previous work [2, 16]. We have, however, included the results of the analysis investigating the impact of medication (using Sackeim scores and chlorpromazine equivalents (CPZ)) in Supplement 2. These analyses reveal a very similar pattern as the main analyses. Third, there are further early risk factors from the epidemiological literature, such as paternal age, or cannabis use which were not included in this study. These factors have mainly been associated with SSD, but not affective disorders, so we did not include them [95]. Fourth, due to the cross-sectional design of this study, no definite inferences can be drawn about causality or directionality.

## Conclusion

Smaller hippocampal GMV may constitute a shared neural pathomechanism in affective and psychotic disorders. We provide multimodal data to associate this finding with psychopathology, neuropsychology, and early and current risk and protective factors. Together with findings from other areas of research, such as genetics, immunology, early and late environmental risks, it emerges that there is an overlap across traditional phenomenological diagnoses, which should be addressed in research using novel approaches that might be better able to acknowledge shared features.

## Supplementary information

Supplementary Material
