# Supplementary material for: Reduced hippocampal gray matter volume is a common feature of patients with major depression, bipolar disorder, and schizophrenia spectrum disorders
Source: Mol Psychiatry. 2022 Jul 15;27(10):4234–43. doi: 10.1038/s41380-022-01687-4 (PMC9718668; doi:10.1038/s41380-022-01687-4)
Supplement: Supplementary file 1 — Supplementary Material [file 41380_2022_1687_MOESM1_ESM.docx]

**Supplement: Reduced hippocampal gray matter volume is a common feature of patients with major depression, bipolar disorder, and schizophrenia spectrum disorders**

**Supplement 1: Neuropsychology factor analysis**

We reduced the neuropsychological test results into intercorrelated domains. The Kaiser-Meyer-Olkin test indicated that the data were appropriate for factor modelling (KMO=.88). Bartlett’s test of sphericity was also significant: *x*^2^=1662.94, df=55, *p*<.001. Exploratory factor analysis with varimax rotation yielded three neuropsychological factors (see eTable1). The three factors were: F1: working memory/executive functioning, F2: verbal fluency, and F3: verbal episodic memory, explaining a total of 50.64% of variance. The factor working memory/executive functioning encompassed the neuropsychological tests of attention (d2), symbol coding, spatial span, letter-number span, and trail-making test (TMT). The second factor *verbal fluency* comprised semantic, lexical, and categorical verbal fluency. The third factor *verbal episodic memory* covered the recognition score German Verbal Learning and Memory Test (VLMT), VLMT verbal episodic memory, and VLMT loss short time delay.

**eFigure 1: Screeplot of explorative factors analysis on neuropsychological tests (matched sample)**


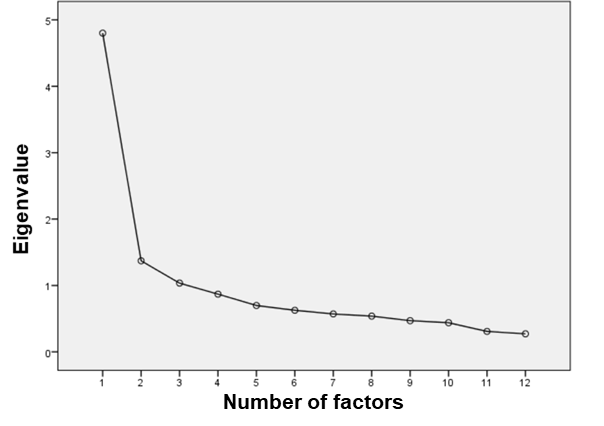


**eTable1: Explorative factor analysis of neuropsychological tests with varimax rotation**

| **Factor** | **Neuropsychological tests** | **Loading** | **Variance explained** |
| --- | --- | --- | --- |
| Working memory/executive functioning | D2 test of attention | .732 | 19.77% |
|  | Symbol coding | .648 |  |
|  | Spatial span | .629 |  |
|  | Letter number span | .506 |  |
|  | TMT difference | -.484 |  |
| Verbal fluency | Semantic verbal fluency | .679 | 15.99% |
|  | Lexical verbal fluency | .672 |  |
|  | Categorical verbal fluency | .595 |  |
| Verbal episodic memory | VLMT recognition | .805 | 14.89% |
|  | VLMT verbal episodic memory | .720 |  |
|  | VLMT loss short time delay | -.403 |  |

**Supplement 2: Additional analyses controlling for medication**

To rule out possible effects of medication on GMV, we performed additional analyses using chlorpromazine equivalent (CPZ) scores and Sackeim scores and correlated these with the identified clusters.

**eTable2: Correlation between conjunction cluster/ F-Test clusters and CPZ Scores, and Sackeim scores**

|  | | **Conjunction Cluster:**  Left Hippocampus | **F-Test Cluster 1:**  Left Fusiform gyrus | **F-Test Cluster 2:** Right Central operculum | **F-Test**  **Cluster 3:**  Left Central/ frontal operculum |
| --- | --- | --- | --- | --- | --- |
| **CPZ score** | *r* | -0.114 | -0.209 | -0.125 | -0.143 |
|  | *p* | 0.041 | <0.001* | 0.025 | 0.010 |
|  | *df* | 323 | 323 | 323 | 323 |
| **Sackeim score** | *r* | -0.073 | -0.017 | 0.016 | -0.008 |
|  | *p* | 0.192 | 0.761 | 0.780 | 0.887 |
|  | *df* | 323 | 323 | 323 | 323 |

**indicates statistical significance at p<.006 corrected for multiple comparisons*

To investigate possible medication effects even further, we performed ANCOVA, to examine for significant differences in GMV in the clusters in patients with and without the intake of antidepressants, antipsychotics, or mood stabilizers.

|  | | **Conjunction Cluster:**  Left Hippocampus | **F-Test  Cluster 1:**  Left Fusiform gyrus | **F-Test  Cluster 2:** Right Central operculum | **F-Test**  **Cluster 3:**  Left Central/ frontal operculum |
| --- | --- | --- | --- | --- | --- |
| **Antidepressant medication**  yes *n*=149  no *n*=181 | *F* | 1.04 | 0.02 | 1.03 | 0.25 |
|  | *p* | 0.308 | 0.908 | 0.311 | 0.615 |
|  | *df* | 323 | 323 | 323 | 323 |
| **Antipsychotic medication**  yes *n*=166  no *n*=164 | *F* | 3.31 | 14.04 | 16.37 | 21.33 |
|  | *p* | 0.070 | <0.001* | <0.001* | <0.001* |
|  | *df* | 323 | 323 | 323 | 323 |
| **Mood stabilizers**  yes *n*=77  no *n*=253 | *F* | 2.03 | 0.001 | 1.84 | 0.16 |
|  | *p* | 0.155 | 0.974 | 0.175 | 0.686 |
|  | *df* | 323 | 323 | 323 | 323 |

**eTable3: ANCOVA conjunction cluster/ F-Test clusters and medication for antidepressants, antipsychotics, and mood stabilizers.**

**indicates statistical significance at p<.004, corrected for multiple comparisons*

**Supplement 3: Results of exploratory whole-brain conjunction analyses**

To add to the ROI-based conjunction analysis (*HC > MDD* $\cap$ *HC > BD* $\cap$ *HC > SSD),* we also investigated this conjunction on a whole-brain level in at *p* < .0001 uncorrected. Compared to HC, MDD, BD, and SSD patients showed consistend reductions in GMV in five clusters.

The first cluster comprised the left hippocampus (25%), left fusiform gyrus (10%), and left parahippocampal gyrus (*k*=636, x/y/z=-30/-33/-10), *T*=4.25, *p*=.127 FWE cluster-level; *p*=.147 FWE peak-level). The second cluster comprised parts of the bilateral gyrus rectus (61%) (*k*=24, x/y/z=0/42/-30), *T*=3.42, *p*=.971 FWE cluster-level; *p*=.903 FWE peak-level). The third cluster includes the right superior temporal gyrus (44%), right middle temporal gyrus (34%), and the right planum polare (20%) (*k*=54, x/y/z=52/-6/-14), *T*=3.37, *p*=.967 FWE cluster-level; *p*=.933 FWE peak-level). The fourth cluster comprised parts of the left middle temporal gyrus (76%) and the left inferior occipital gyrus (16%) (*k*=27, x/y/z=-54/-64/2), *T*=3.37, *p*=.967 FWE cluster-level; *p*=.933 FWE peak-level). Finally, the fifth cluster included parts of the left operculum (44%), left anterior insula (28%), left frontal operculum (13%), and inferior frontal gyrus (10%) (*k*=22, x/y/z=-44/4/4), *T*=3.21, *p*=.973 FWE cluster-level; *p*=.984 FWE peak-level).

**Supplement 4*:* Comorbidities in the patient groups**

Regarding **comorbid conditions,** MDD, BD, SSD did not differ in the presence of comorbidity (x²(1,2)=1.58, p=.453). Within MDD patients, *n*=36 had at least one comorbidity, within BD patients *n*=44, and within SSD patients *n*=41. Please see the following table for a detailed overview of all comorbidities.

***eTable4: Number of DSM-IV comorbid disorders in patient groups***

| ***Disorder*** | ***Total*** | ***MDD*** | ***BD*** | ***SSD*** |
| --- | --- | --- | --- | --- |
| *Anxiety disorders* | *74* | *26* | *28* | *20* |
| *Dysthymia* | *14* | *14* | *-* | *-* |
| *Obsessive compulsive disorder* | *20* | *8* | *7* | *5* |
| *Eating disorders* | *18* | *2* | *7* | *9* |
| *Alcohol abuse* | *23* | *4* | *10* | *9* |
| *Substance abuse* | *25* | *2* | *3* | *20* |
| *Pain disorder* | *1* | *1* | *-* | *-* |
| *Body dysmorphic disorder* | *1* | *-* | *1* | *-* |
| *Post-traumatic stress disorder* | *12* | *1* | *6* | *5* |
| ***SUM*** | ***188*** | ***58*** | ***62*** | ***68*** |

*Note: Current or life-time alcohol and substance dependency (other than cannabis) constituted exclusion criteria.*

As patient groups did not significantly differ in the presence of comorbidity, we did not include this as a covariate to the statistical models. However, to exclude a potential effect of comorbidity, we performed post-hoc ANCOVA analyses by extracting eigenvariates (weighted mean) of significant clusters and then compared mean values between patients with and without comorbidity, controlling for relevant covariates. Results indicated no difference between patients comorbidity in the detected GMV clusters:

***eTable5: Differences in GMV clusters in patients with and without comorbidities***

|  | | **Conjunction Cluster:**  Left Hippocampus | **F-Test Cluster 1:**  Left Fusiform gyrus | **F-Test Cluster 2:** Right Central operculum | **F-Test Cluster 3:**  Left Central/ frontal operculum |
| --- | --- | --- | --- | --- | --- |
| **Comorbidity**  yes *n*=121  no *n*=209 | *F* | 0.99 | 2.7 | 3.03 | 3.04 |
|  | *p* | 0.320 | 0.101 | 0.083 | 0.082 |
|  | *df* | 322 | 322 | 322 | 322 |

**Supplement 5: Additional analyses: *n*=330 HCs compared to *n*=330 patients**

To test the robustness of our main findings with regard to the HC group, we re-ran the analyses adding *n*=220 HCs matched for age and sex to the three patient groups.

1. *Global effects of GMV (F-Test):*

For the four groups (HC, MDD, BD, SSD), five significant clusters emerged in the *F*-Test. The first cluster comprised parts of the left right middle and superior temporal gyri (*k*=18304, x/y/z=62/-39/2), *F*=15.04, *p*<.0001 FWE cluster-level; *k*=3028, *p*<.0001 FWE peak-level). The second cluster comprised the left fusiform and inferior temporal gyri (*k*=161167, x/y/z=-38/-33/-22), *F*=14.21, *p*<.0001 FWE cluster-level; *k*=264, *p*<.0001 FWE peak-level). The third cluster comprised parts of the bilateral thalami (*k*=1845, x/y/z=-3/-9/10), *F*=12.13, *p*=.005 FWE cluster-level; *k*=485, *p*=.002 FWE peak-level). The fourth cluster included the right gyrus rectus and bilateral anterior cingulate gyri (*k*=3130, x/y/z=8/28/-15), *F*=10.18, *p*<.0001 FWE cluster-level; *k*=88, *p*=.009 FWE peak-level). The fifth cluster encompassed the right supramarginal and angular gyri (*k*=1084, x/y/z=50/-42/51), *F*=10.59, *p*=.04 FWE cluster-level; *k*=179, *p*=.011 FWE peak-level).

***eFigure2: F-Test for N=440 sample (left) and N=660 sample (right)***

*
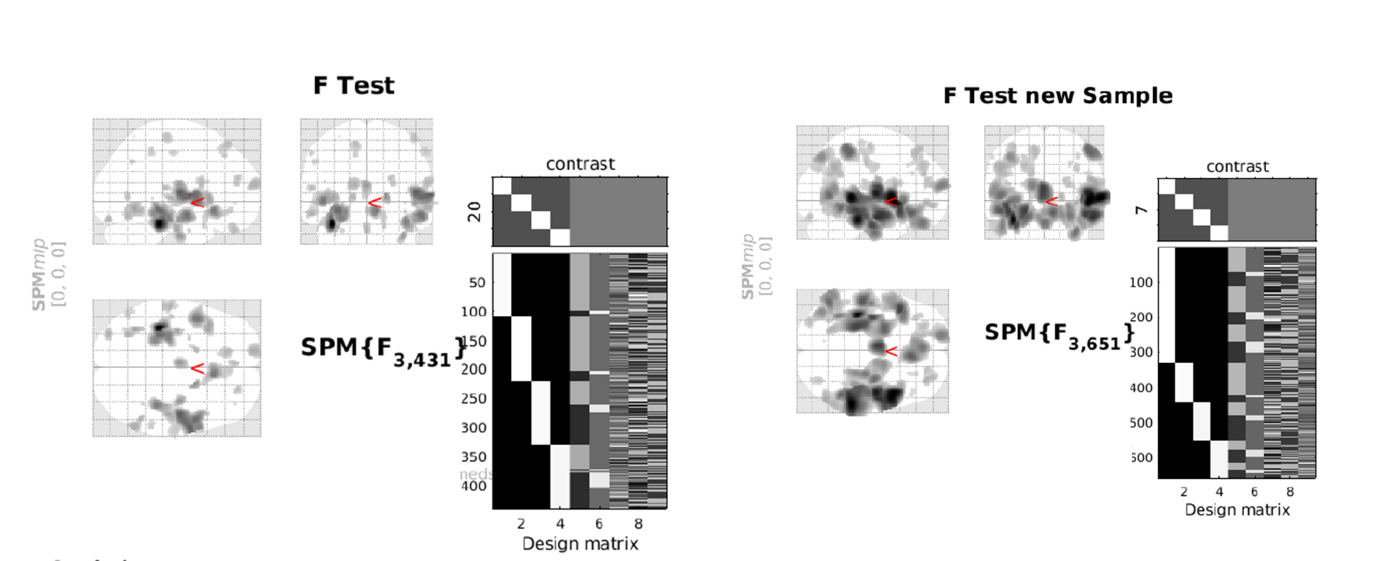
*

1. *Investigation of post-hoc two-group comparisons of GMV (t-Tests)*

The eTable6 below gives a detailed overview of post-hoc two-group comparisons. These results align with the results of the original analyses using a 1:1 matching.

***eTable 6:* *Post-hoc two-group comparisons of GMV (t-Tests) in N=660 subjects***

|  |  | MNI coordinates | | |  |  |  |  |
| --- | --- | --- | --- | --- | --- | --- | --- | --- |
|  | **H** | **x** | **y** | **z** | ***t*** | ***k***  **cluster** | ***p* FWE cluster level** |  |
| **HC vs. diagnostic group** | | | | | | | |  |
| **HC > MDD** | | | | | | | |  |
| 45% frontal pole  39% superior frontal gyrus | L | -15 | 64 | 15 | 4.58 | 1181 | .071 |  |
| 39% parahippocampal gyrus  17% hippocampus  17% fusiform gyrus | L | -24 | -34 | -14 | 4.37 | 3009 | .003 |  |
| 40% temporal transverse gyrus  31% central operculum  19% posterior insula | R | 46 | -14 | 8 | 4.14 | 2310 | .008 |  |
| **HC > BD** | | | | | | | |  |
| 69% middle temporal gyrus  16% inferior temporal gyrus | L | -51 | -27 | -12 | 4.92 | 1169 | .072 |  |
| 46% middle temporal gyrus  16% superior temporal gyrus | R | 46 | -36 | 2 | 4.87 | 3645 | .001 |  |
| 52% middle temporal gyrus  22% inferior occipital gyrus  13% angular gyrus | L | -44 | -64 | 9 | 4.52 | 1205 | .067 |  |
| **HC > SSD** | | | | | | | |  |
| 37% central operculum  16% inferior frontal gyrus  14% frontal operculum  13% planum polare | R | 51 | 6 | 3 | 6.47 | 26042 | <.0001 |  |
|  | | | | | | | |  |
| 79% fusiform gyrus | L | -36 | -33 | -21 | 6.27 | 28341 | <.0001 |  |
|  | | | | | | | |  |
| 57% thalamus proper | B | -3 | -9 | 10 | 5.9 | 3027 | .003 |  |
|  | | | | | | | |  |
| 27% gyrus rectus  23% medial orbital gyrus  12% anterior cingulate gyrus | R | 8 | 30 | -16 | 4.91 | 7061 | <.0001 |  |
| 41% angular gyrus  36% supramarginal gyurs | R | 50 | -48 | 56 | 4.92 | 2071 | .013 |  |
| 58% postcentral gyrus  27% precentral gyrus | L | -36 | -22 | 39 | 4.52 | 3631 | .001 |  |
| 44% anterior orbital gyrus  29% middle frontal gyrus  24% lateral orbital gyrus | R | 32 | 52 | -8 | 4.34 | 2075 | .013 |  |
|  |  |  |  |  |  |  |  |  |
| *eTable 6* *continued.*  **Differences between diagnostic groups** | | | | | | | |  |
| **MDD < BD** | | | | | | | |  |
| 40% frontal pole  28% superior frontal gyrus | L | -16 | 68 | 16 | 4.49 | 662 | .22 |  |
| **MDD > SSD** | | | | | | | | |
| 79% fusiform gyrus  11% inferior temporal gyrus | L | -37.5 | -33 | -24 | 5.56 | 1628 | .029 |  |
| 74% fusiform gyrus  11% parahippocampal gyrus  11% inferior temporal gyrus | R | 34 | -13.5 | -36 | 4.76 | 2275 | .009 |  |
| **BD > SSD** | | | | | | | | |
| 80% fusiform gyrus  11% inferior temporal gyrus | L | -38 | -33 | -22 | 4.97 | 1668 | .027 |  |
| 85% fusiform gyrus | R | 38 | -38 | -21 | 4.77 | 1915 | .017 |  |
| 38% anterior cingulate gyrus  12% gyrus rectus | R | 6 | 24 | -12 | 4.45 | 1868 | .019 |  |

*Note*: H=hemisphere, R=right, L=left, B=bilateral. Only areas $\geq$ 10% are included. Letters indicate clusters which were also significant at FWE peak level.

1. *Common areas of GMV alterations across MDD, BD, and SZ*

To investigate if results of commonly reduced GMV in MDD, BD, and SSD were driven by the matched HC group, we re-ran analyses adding *n*=220 additional healthy controls (HC) matched for age and sex to the original sample. Confirmatory conjunction analysis (*HC > MDD* $\cap$ *HC > BD* $\cap$ *HC > SSD)* using the ROIs from the meta-analysis by Goodkind et al., (2015) revealed diagnosis-commonly reduced hippocampal GMV in this sample, too (*k*=260, x/y/z=-26/-30/-14), *T*=4.06, *p*=.022 FWE cluster level; k=260, *p*=.004 FWE peak-level). In addition, we performed exploratory (p<.0001, uncorrected) whole-brain conjunction analysis. Hereof, nine clusters can be identified, that are commonly reduced in MDD, BD, and SSD patients compared to healthy subjects at p<.0001, uncorrected:

Cluster 1: left hippocampus (28%) and parahippocampal gyrus (41%) (*k*=559, x/y/z=-26/-30/-14), *T*=4.06, *p*=.279 FWE cluster-level; *p*=.167 FWE peak-level.

Cluster 2: right middle (81%) temporal and superior (19%) temporal gyri (*k*=1173, x/y/z=63/-39/2), *T*=3.85, *p*=.072 FWE cluster-level; *p*=.318 FWE peak-level.

Cluster 3: right planum polare (38%), superior temporal gyrus (22%), posterior insula (15), and right middle temporal gyrus (15%) (*k*=102, x/y/z=45/-8/-14), *T*=3.51, *p*=.793 FWE cluster-level; *p*=.667 FWE peak-level.

Cluster 4: left middle temporal gyrus (54%) (*k*=139, x/y/z=-62/-64/6), *T*=3.46, *p*=.735 FWE cluster-level; *p*=.723 FWE peak-level.

Cluster 5: right hippocampus (42%) and parahippocampal gyrus (36%) (*k*=220, x/y/z=27/-22/-16), *T*=3.4, *p*=.616 FWE cluster-level; *p*=.782 FWE peak-level.

Cluster 6: bilateral gyrus rectus (*k*=77, x/y/z=0/40/-30), *T*=3.42, *p*=.833 FWE cluster-level; *p*=.757 FWE peak-level.

Cluster 7: right middle (76%) and superior (23%) temporal gyri (*k*=37, x/y/z=56/-6/-20), *T*=3.42, *p*=.897 FWE cluster-level; *p*=.876 FWE peak-level.

Cluster 8: right anterior insula (33%), right temporal pole (30%), and right frontal operculum (16%) - *k*=44, x/y/z=46/10/-8), *T*=3.27, *p*=.886 FWE cluster-level; *p*=.885 FWE peak-level.

Cluster 9: right anterior insula (41%) and right temporal pole (38%) (*k*=13, x/y/z=42/15/14), *T*=3.21, *p*=.936 FWE cluster-level; *p*=.921 FWE peak-level.
